# An open‐label, dose‐escalation study to evaluate the safety, tolerability, pharmacokinetics, and pharmacodynamics of single doses of GSK2586881 in participants with pulmonary arterial hypertension

**DOI:** 10.1002/pul2.12024

**Published:** 2022-01-20

**Authors:** Marc A. Simon, Kate Hanrott, David C. Budd, Fernando Torres, Ekkehard Grünig, Pilar Escribano‐Subias, Manuel L. Meseguer, Michael Halank, Christian Opitz, David A. Hall, Deborah Hewens, William M. Powley, Sarah Siederer, Andrew Bayliffe, Aili L. Lazaar, Anthony Cahn, Stephan Rosenkranz

**Affiliations:** ^1^ Division of Cardiology, Department of Medicine University of California San Francisco California USA; ^2^ Research and Development, Medicines Research Centre GlaxoSmithKline plc. Stevenage UK; ^3^ UT Southwestern Medical Center Dallas Texas USA; ^4^ Centre for Pulmonary Hypertension Thoraxklinik Heidelberg gGmbH at Heidelberg University Hospital Heidelberg Germany; ^5^ CIBER‐CV Cardiology Department, Pulmonary Hypertension Unit Hospital Universitario 12 de Octubre Madrid Spain; ^6^ Lung Transplant and Pulmonary Vascular Diseases Department Hospital Universitari Vall d'Hebron Barcelona Spain; ^7^ Department of Internal Medicine I University Hospital Carl Gustav Carus Dresden Germany; ^8^ Department of Cardiology DRK Kliniken Berlin Germany; ^9^ Department of Cardiology, University Heart Center Berlin Charité University Medicine Berlin Germany; ^10^ Marengo Therapeutics and Apple Tree Partners Cambridge Massachusetts USA; ^11^ Discovery Medicine, Clinical Pharmacology and Experimental Medicine GlaxoSmithKline plc. Collegeville Pennsylvania USA; ^12^ Department III of Internal Medicine, Cologne Cardiovascular Research Center (CCRC) Cologne University Heart Center Cologne Germany

**Keywords:** arterial hypertension, hemodynamics, recombinant human angiotensin‐converting enzyme 2, renin‐angiotensin system, rhACE2

## Abstract

Preclinical and early clinical studies suggest that angiotensin‐converting enzyme type 2 activity may be impaired in patients with pulmonary arterial hypertension (PAH); therefore, administration of exogenous angiotensin‐converting enzyme type 2 (ACE2) may be beneficial. This Phase IIa, multi‐center, open‐label, exploratory, single‐dose, dose‐escalation study (NCT03177603) assessed the potential vasodilatory effects of single doses of GSK2586881 (a recombinant human ACE2) on acute cardiopulmonary hemodynamics in hemodynamically stable adults with documented PAH who were receiving background PAH therapy. Successive cohorts of participants were administered a single intravenous dose of GSK2586881 of 0.1, 0.2, 0.4, or 0.8 mg/kg. Dose escalation occurred after four or more participants per cohort were dosed and a review of safety, tolerability, pharmacokinetics, and hemodynamic data up to 24 h postdose was undertaken. The primary endpoint was a change in cardiopulmonary hemodynamics (pulmonary vascular resistance, cardiac index, and mean pulmonary artery pressure) from baseline. Secondary/exploratory objectives included safety and tolerability, effect on renin‐angiotensin system peptides, and pharmacokinetics. GSK2586881 demonstrated no consistent or sustained effect on acute cardiopulmonary hemodynamics in participants with PAH receiving background PAH therapy (*N* = 23). All doses of GSK2586881 were well tolerated. GSK2586881 was quantifiable in plasma for up to 4 h poststart of infusion in all participants and caused a consistent and sustained reduction in angiotensin II and a corresponding increase in angiotensin (1–7) and angiotensin (1–5). While there does not appear to be a consistent acute vasodilatory response to single doses of GSK2586881 in participants with PAH, the potential benefits in terms of chronic vascular remodeling remain to be determined.

## INTRODUCTION

Pulmonary arterial hypertension (PAH) is a progressive condition^1^ characterized by enhanced pulmonary vasoconstriction, small pulmonary arterial remodeling, and loss of vessel compliance/distensibility.^1^, ^2^, ^3^, ^4^ These changes increase pulmonary vascular resistance (PVR) and subsequently pulmonary arterial pressure (PAP) and cause right ventricular failure leading to premature death.^1^, ^5^, ^6^ In the last two decades, several new treatments have become available for PAH, including those that specifically target endothelial dysfunction.^7^ Therapies specifically for PAH in the current treatment landscape have improved outcomes in PAH, but overall long‐term prognosis remains poor and mortality remains high.^5^ The mainstay of the standard of care for PAH is treatment with vasodilators; however, these drugs mainly improve symptoms and exercise capacity and do not influence disease progression.^8^ Standard of care treatments are relatively ineffective at impacting vascular remodeling,^9^ despite a substantial proportion of patients with PAH displaying vascular remodeling.^10^ There is, therefore, a need for new, more effective therapies that attenuate the pathological mechanisms that drive vascular remodeling.^9^

**Figure 1:**
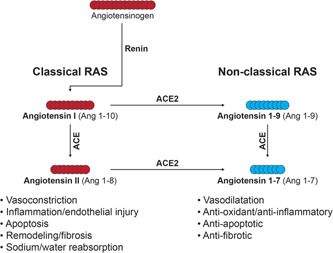
Overview of the renin‐angiotensin system pathway. ACE(2), angiotensin‐converting enzyme (2); Ang, angiotensin; RAS, renin‐angiotensin system

Clinical studies have shown that the tissue renin‐angiotensin system (RAS) becomes dysregulated in the remodeled arterial walls in the lungs of individuals with PAH.^11^, ^12^ Angiotensin II (Ang II) is a key effector peptide of the RAS (Figure 1) that can exert deleterious effects on the pulmonary vasculature, resulting in vasoconstriction via modulation of pulmonary vessel tone, inflammation, proliferation, and structural remodeling, which contribute to PAH development.^1^, ^2^, ^3^, ^4^, ^13^, ^14^, ^15^ Angiotensin‐converting enzyme type 2 (ACE2) is a type I, membrane‐bound carboxypeptidase that inactivates Ang II by cleaving it to form Ang(1–7). Ang(1–7) has been shown to have vasodilatory, antiproliferative, and anti‐inflammatory properties in experimental models.^15^, ^16^ In rodent models of PAH, recombinant human ACE2 has been shown to treat established PAH, including bone morphogenetic protein receptor type II mutation‐related PAH^17^ and pulmonary artery banding models.^18^ Systemic and pulmonary Ang II levels have been reported to be elevated in patients with idiopathic PAH and are associated with increased pulmonary vascular remodeling,^11^ while serum ACE2 levels are reported to be decreased in patients with congenital heart disease‐associated PAH and correlate with mean PAP (mPAP).^19^ The presence of autoantibodies to ACE2 has also been reported in the serum of patients with autoimmune disease with constrictive vasculopathies, including PAH, and correlates with reduced ACE2 activity.^20^ Taken together, these studies suggest that ACE2 activity may be impaired in patients with PAH and administration of exogenous ACE2 may be beneficial.

GSK2586881 is a recombinant version of the catalytic ectodomain of recombinant human ACE2. In healthy participants (NCT00886353)^21^ and mechanically ventilated patients with acute respiratory distress syndrome (NCT01597635),^22^ GSK2586881 was well tolerated with rapid modulation of RAS peptides, suggesting target engagement. In a single‐center, dose‐escalation pilot study in which five PAH patients were administered a single intravenous (IV) dose of GSK2586881 (0.2–0.4 mg/kg), there was a significant increase in cardiac output and a reduction in PVR (NCT01884051).^23^ The aim of this study was to test the safety and effects on cardiopulmonary hemodynamics of single doses of GSK2586881 in participants with PAH receiving background PAH therapy.

## METHODS

### Objectives

The primary objective was to evaluate the effect of single IV doses of GSK2586881 on changes in cardiopulmonary hemodynamics (PVR, cardiac index [CI], and mPAP) of participants with PAH receiving one or more background PAH therapies. Secondary/exploratory objectives were to evaluate the following of single IV doses of GSK2586881: (1) safety and tolerability, (2) effect on RAS peptides, (3) effect on biomarkers of disease activity (N‐terminal pro‐B‐type natriuretic peptide [NT‐proBNP] and nitrogen oxyanions [nitrate, nitrite, and their sum NO_*x*_^–^], and (4) pharmacokinetics (PK).

### Study design

We conducted a Phase IIa, multicenter, open‐label, exploratory, dose‐escalation study at eight centers (four in Germany, two in Spain, and two in the United States) from February 2018 to May 2019 (GlaxoSmithKline plc. study 206246; NCT03177603). The study consisted of a screening period (up to 28 days before dosing), dosing, a 24 h observation period after the single dose, and two follow‐up visits (at 7–14 days postdose and at approximately 28 days postdose). Successive cohorts of participants (minimum of four participants per cohort) were given a single IV dose of GSK2586881 of 0.1, 0.2, 0.4, or 0.8 mg/kg. The dose for the initial cohort was 0.1 mg/kg. Dose escalation to 0.2, 0.4, and a maximum dose of 0.8 mg/kg occurred after a minimum of four participants per cohort were dosed and a review of safety, PK, and hemodynamic data up to 24 h postdose was undertaken.

### Participants

The study population comprised hemodynamically stable adults aged 18–75 years with an established and documented diagnosis of PAH (mPAP >25 mmHg and pulmonary arterial wedge pressure ≤15 mmHg). Participants had a systemic mean arterial pressure >60 mmHg and a 6‐min walk distance (6MWD) at screening (or within 6 months of screening) of >100 and <500 m. Full inclusion and exclusion criteria are available at clinicaltrials.gov (NCT03177603).

### Endpoints

The primary endpoint of the study was a change in cardiopulmonary hemodynamics from baseline, as assessed by PVR, CI, and mPAP. Secondary endpoints were: (1) safety: effect of GSK2586881 on adverse events (AEs), clinical laboratory values, vital signs (systolic and diastolic blood pressure, pulse rate, and respiratory rate), 12‐lead electrocardiograms (ECGs), pulse oximetry, and immunogenicity; (2) change from baseline in systemic and pulmonary wedge RAS peptides (Ang II, Ang[1–7], Ang[1–5], and Ang II/Ang[1–7] ratio); (3) change from baseline in NT‐proBNP and NO_*x*_^–^; and (4) plasma concentrations of GSK2586881, and derived PK parameters.

Pulmonary arterial catheters were used to measure cardiopulmonary hemodynamics at four time‐points (predose, and 1, 2, and 4 h postdose). Safety assessments included monitoring of vital signs, 12‐lead ECGs, clinical laboratory tests, physical examinations, and the number and type of AEs, all of which were performed at various time‐points between study screening until final follow‐up at 7–14 days after the last dose. PK assessment of GSK2586881 by immunoassay, and pharmacodynamic/biomarker assessments of RAS peptides (Ang II, Ang[1–7], Ang[1–5], and Ang II/Ang[1–7] ratio) by a method based on reversed‐phase solid‐phase extraction and assessment of disease activity biomarkers (NT‐proBNP and NO_*x*_^−^), were performed as previously described.^22^ A detailed schedule of assessments is shown in Table S1.

Across the observation period (from predose to 24 h postdose), PK and biomarker assessments of disease activity were performed at eight and four time‐points, respectively. Pharmacodynamic/biomarker assessments of systemic and pulmonary wedge RAS peptides were performed at four and eight time‐points during the observation period, respectively, and systemic biomarkers were also assessed once at follow‐up (at 7–14 days postdose).

The evaluable population, defined as all participants who received at least one dose of GSK2586881 who completed all assessments during the observation period and who did not have major protocol deviations, was the primary population for pharmacodynamic analyses. The safety and PK populations included all participants who received at least one dose of GSK2586881.

### Statistical methods

Determination of sample size was not based on statistical considerations. On the basis of previous clinical experience with GSK2586881, a minimum of four participants were planned per cohort, with a maximum of 27 enrolled participants. No formal statistical hypotheses were tested in the study, and all data were summarized descriptively. For the primary endpoints, pulmonary hemodynamics, Bayesian repeated‐measures mixed‐effect models (one model per endpoint) were used to assess changes from baseline using log‐transformed data for dose group and posttreatment time‐point combinations. Posterior distributions were used to assess probability statements for individual pulmonary hemodynamic parameters, for example, the probability of a decrease from baseline in PVR of >15% (i.e., a ratio to baseline <0.85). Events with a high probability of occurring (i.e., values close to 1) were of interest. Details on the final planned primary analyses are provided in the Supporting Information Methods.

A posthoc responder analysis of individual participant data was performed for the primary endpoints PVR and CI. A response was defined as a decrease in PVR of >15% with an increase in CI of >6% at 1 h postdose. These values were based on a study in which the vasodilatory effects of sildenafil were investigated in PAH patients receiving standard of care.^24^ Plasma GSK2586881 concentration–time data were analyzed by noncompartmental methods using Phoenix WinNonlin 8.1.

Further analysis on the effect of GSK2586881 on Ang II/Ang(1–7) ratio, and on mPAP, CI, and PVR by dose and PAH type (idiopathic, heritable, associated with collagen vascular disease) is also presented.

## RESULTS

### Study population

**Figure 2:**
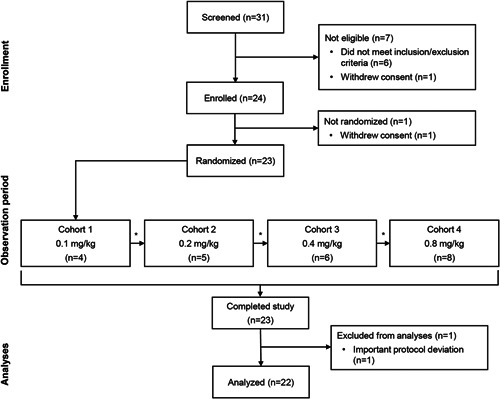
Participant treatment flow. *Dose‐escalation meeting (review of safety, pharmacokinetics, and hemodynamic data). One participant was excluded from the evaluable analysis population due to a protocol violation (taking a prohibited medication [an angiotensin II receptor antagonist]). This participant was included in the safety and pharmacokinetics analyses

Of 31 participants screened, 23 were randomized to treatment (six participants were excluded for not meeting the eligibility criteria, and two withdrew consent) (Figure 2). One patient was excluded from the evaluable population due to a protocol deviation (taking a prohibited medication [an Ang II receptor antagonist]). All participants received their intended dose, except one participant in Cohort 4 (0.8 mg/kg) who received 0.67 mg/kg. Demographics and baseline characteristics of the participants were similar across cohorts, except for Cohort 1 (0.1 mg/kg), which had a lower mean age and body mass index than the other three cohorts (Table 1). Participants were all in World Health Organization functional class II (*n* = 14/23) or III (*n* = 9/23). Mean 6MWD across all cohorts was 416 m and was approximately consistent across the cohorts, except for Cohort 2 (0.2 mg/kg), which was shorter than average (340 m). The majority of participants (*n* = 12/23) had idiopathic PAH. Most patients received double (*n* = 8/23) or triple (*n* = 12/23) background PAH therapy at baseline. Concomitant medications are detailed in Table S2.

**Table 1:** Participant demographics and baseline characteristics by GSK2586881 cohort

	Cohort 1 GSK2586881 0.1 mg/kg (*n* = 4)	Cohort 2 GSK2586881 0.2 mg/kg (*n* = 5)	Cohort 3 GSK2586881 0.4 mg/kg (*n* = 6)	Cohort 4 GSK2586881 0.8 mg/kg (*n* = 8)	Total (*N* = 23)
Age (years), mean (range)^a^	32 (26–38)	55 (36–74)	53 (45–67)	50 (23–67)	49 (23–74)
Female (*n*)	3	3	4	5	15
BMI (kg/m^2^), mean (range)	20.7 (18.6–22.6)	26.8 (25.7–29.7)	28.9 (22.0–32.9)	28.5 (21.0–34.6)	26.9 (18.6–34.6)
WHO functional class (*n*)					
II	3	3	4	4	14
III	1	2	2	4	9
Six‐minute walk distance, (m), mean (range)	434 (405–446)	340 (254–409)	439 (340–498)	437 (347–495)	416 (254–498)
PAH type underlying reason (*n*)					
Idiopathic PAH	3	3	3	3	12
Heritable PAH	1	0	1	4	6
PAH associated with collagen vascular disease	0	2	2	1	5
Concomitant medications for PAH					
Monotherapy	0	1	0	0	1
Dual therapy	1	1	1	5	8
Triple therapy	3	3	4	2	12

Abbreviations: BMI, body mass index; PAH, pulmonary arterial hypertension; WHO, World Health Organization.

^a^ Birth date was imputed as June 30 in the year of birth.

### Cardiopulmonary hemodynamics (PVR, CI, and mPAP)

**Figure 3:**
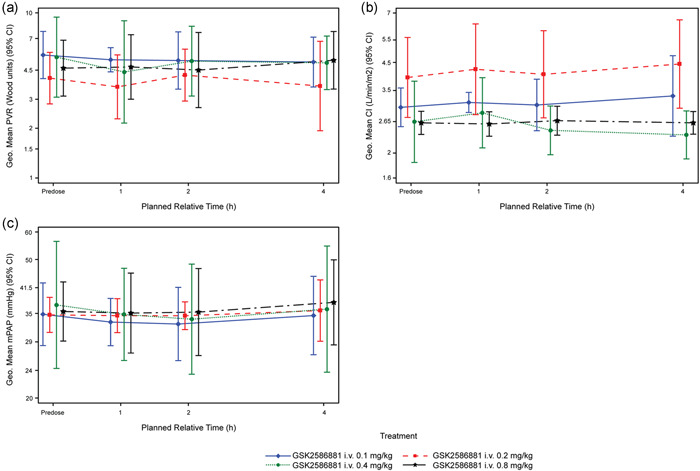
Hemodynamics following treatment with GSK2586881. Geometric mean time profiles and 95% confidence intervals (denoted as 95% CI; evaluable population) for (a) PVR, (b) CI, and (c) mPAP. CI, cardiac index; i.v., intravenous; mPAP, mean pulmonary artery pressure; PVR, pulmonary vascular resistance

Changes in cardiopulmonary hemodynamics are summarized in Table 2. Across all cohorts, no postdose changes in mean PVR, CI, or mPAP were observed following administration of a single IV dose of GSK2586881 (0.1, 0.2, 0.4, and 0.8 mg/kg) (Figure 3a–c). Posterior probabilities for decreases from baseline in PVR were low at all time‐points, across all dose levels. The probability of a >15% decrease in PVR (ratio <0.85) at 1 h postdose was 68% for Cohort 3 (0.4 mg/kg) and <35% for other cohorts (Table S3). Posterior probabilities for increases from baseline in CI were low at most time‐points and dose levels. However, in Cohort 2 (GSK2586881 0.2 mg/kg), the probability of a >6% increase in CI at 1 h postdose was 86%, and at 4 h was 95% (Table S3). This was due to one participant having a sharp increase in CI at 4 h. Additionally, no concurrent decrease in PVR and mPAP at 1 or 4 h postdose was observed in this participant, so results are unlikely to be clinically relevant. Posterior probabilities for decreases from baseline in mPAP were low at most time‐points and dose levels. The probability of a >10% decrease in mPAP from baseline was <44% at all dose levels and time‐points (data not shown).

**Table 2:** Summary of cardiopulmonary hemodynamics by GSK2586881 cohort

	Cohort 1 GSK2586881 0.1 mg/kg (*n* = 4)				Cohort 2 GSK2586881 0.2 mg/kg (*n* = 5)				Cohort 3 GSK2586881 0.4 mg/kg (*n* = 6)				Cohort 4 GSK2586881 0.8 mg/kg (*n* = 8)			
Parameter	Predose	1 h postdose	2 h postdose	4 h postdose	Predose	1 h postdose	2 h postdose	4 h postdose	Predose	1 h postdose	2 h postdose	4 h postdose	Predose	1 h postdose	2 h postdose	4 h postdose
PASP (mmHg)	57.2 (46.9, 69.7)	57.4 (51.1, 64.4)	58.1 (44.2, 76.3)	60.6 (49.5, 74.2)	60.1 (52.4, 68.9)	60.3 (52.0, 69.8)	60.5 (52.4, 69.9)	61.8 (49.6, 77.1)	59.7 (38.5, 92.5)	55.2 (37.2, 81.9)	55.0 (37.5, 80.7)	58.0 (38.8, 86.6)	61.5 (49.4, 76.7)	62.9 (48.7, 81.2)	60.7 (44.4, 83.1)	64.7 (50.5, 82.8)
PADP (mmHg)	23.6 (18.5, 30.0)	20.7 (14.6, 29.1)	19.6 (13.5, 28.5)	21.2 (13.8, 32.5)	21.9 (19.4, 24.8)	21.6 (20.0, 23.3)	21.4 (20.0, 22.8)	22.6 (18.5, 27.5)	25.1 (15.3, 41.0)	24.1 (17.5, 33.2)	22.9 (15.8, 33.2)	24.9 (15.9, 39.1)	22.3 (18.6, 26.7)	21.1 (15.8, 28.1)	22.4 (16.8, 29.8)	24.0 (17.1, 33.7)
mPAP (mmHg)	34.8 (28.3, 42.8)	33.1 (28.3, 38.6)	32.6 (25.6, 41.6)	34.5 (26.6, 44.7)	34.7 (30.9, 38.9)	34.5 (30.8, 38.6)	34.5 (31.5, 37.8)	35.7 (29.1, 43.7)	37.0 (24.3, 56.4)	34.8 (25.6, 47.2)	33.7 (23.4, 48.5)	36.0 (23.7, 54.7)	35.5 (29.2, 43.1)	35.1 (26.9, 45.7)	35.3 (26.5, 47.1)	37.7 (28.4, 49.9)
PAWP (mmHg)	7.4 (5.2, 10.4)	6.2 (3.7, 10.5)	6.2 (3.4, 11.2)	6.8 (3.4, 13.4)	6.2 (3.6, 10.6)	5.7 (1.7, 19.6)	5.3 (3.2, 8.8)	6.9 (5.1, 9.1)	6.0 (2.1, 17.0)	7.5 (3.7, 15.3)	6.9 (3.2, 14.9)	9.8 (3.6, 26.3)	10.4 (8.7, 12.5)	10.2 (8.9, 11.8)	10.4 (8.5, 12.7)	10.0 (7.6, 13.0)
CO (L/min)	4.9 (4.1, 5.9)	5.1 (5.1, 5.2)	5.0 (4.2, 6.0)	5.5 (4.0, 7.5)	6.9 (5.0, 9.5)	7.5 (5.2, 10.8)	7.1 (5.0, 10.1)	7.9 (5.2, 11.9)	5.4 (3.8, 7.7)	5.8 (4.3, 7.9)	5.0 (4.2, 6.0)	4.8 (3.9, 5.8)	5.1 (4.7, 5.6)	5.1 (4.6, 5.6)	5.2 (4.6, 5.8)	5.1 (4.7, 5.6)
CI (L/min/m^2^)	3.0 (2.5, 3.6)	3.2 (2.9, 3.4)	3.1 (2.4, 3.9)	3.3 (2.3, 4.8)	3.9 (2.8, 5.6)	4.2 (2.8, 6.4)	4.1 (2.7, 6.0)	4.4 (3.0, 6.6)	2.7 (1.8, 3.8)	2.9 (2.1, 3.9)	2.5 (2.0, 3.1)	2.4 (1.9, 2.9)	2.6 (2.4, 2.9)	2.6 (2.3, 2.9)	2.7 (2.4, 3.0)	2.6 (2.4, 2.9)
PVR (Wood units)	5.6 (4.0, 7.7)	5.2 (4.4, 6.2)	5.2 (3.5, 7.7)	5.0 (3.6, 7.1)	4.0 (2.8, 5.8)	3.6 (2.3, 5.6)	4.2 (2.9, 6.0)	3.6 (1.9, 6.7)	5.4 (3.1, 9.4)	4.4 (2.2, 9.0)	5.1 (3.1, 8.3)	5.0 (3.4, 7.2)	4.6 (3.1, 6.8)	4.7 (3.0, 7.4)	4.5 (2.7, 7.6)	5.2 (3.5, 7.7)
RAP (mmHg)	3.8 (1.6, 9.2)	2.2 (0.5, 9.8)	4.4 (3.2, 6.1)	2.0 (0.6, 7.1)	3.3 (1.4, 7.8)	3.5 (2.7, 4.5)	3.2 (2.6, 4.1)	2.6 (1.9, 3.4)	4.0 (0.7, 22.4)	3.3 (0.8, 13.7)	4.0 (1.3, 12.8)	5.1 (1.0, 25.0)	6.0 (4.3, 8.3)	4.6 (2.7, 7.6)	4.4 (2.5, 7.8)	4.6 (2.7, 8.1)

*Note*: Data are geometric mean (95% confidence interval; evaluable population).

Abbreviations: CI, cardiac index; CO, cardiac output; mPAP, mean pulmonary artery pressure; PADP, pulmonary artery diastolic pressure; PASP, pulmonary artery systolic pressure; PAWP, pulmonary arterial wedge pressure; PVR, pulmonary vascular resistance; RAP, right atrial pressure.

Overall, there were no clinically relevant posttreatment changes from baseline in mean cardiopulmonary hemodynamics following administration of a single IV dose of GSK2586881. At 1 h postdose, 20%–40% of participants met the posthoc responder definition in the first three cohorts (*n* = 1/4, 2/5, and 1/5 in the 0.1, 0.2, and 0.4 mg/kg cohorts, respectively); no participants at the highest dose level (0.8 mg/kg) met the definition. No participants demonstrated a sustained response at all time‐points. Of the seven participants who met the response criteria at one or more time‐points, none were receiving mono, three were receiving dual, and four were receiving triple background PAH therapy. There was no apparent correlation between background PAH therapy and response to GSK2586881.

### AEs, clinical laboratory values, vital signs, 12‐lead ECGs, pulse oximetry, and immunogenicity

A total of 11 AEs were reported by 11/23 participants (Table 3). All AEs were mild or moderate in intensity with the exception of one severe AE of back pain, which resolved and was not considered related to study treatment. Two AEs were considered drug‐related: a mild headache and mild erythema of the face, both of which resolved. No AEs led to participant withdrawal from the study, and no serious AEs (SAEs), deaths, or other clinically relevant AEs were reported. Mean changes from baseline in clinical laboratory (chemistry and hematology) values, vital signs (systolic and diastolic blood pressure, heart rate, and respiration rate), and 12‐lead ECGs were minimal in most cases across dose groups, and no trends or changes of clinical concern were identified (data not shown). The majority of ECG values of clinical interest were long PR and QRS intervals in some participants (pre‐ and postdose). One participant in Cohort 3 (0.4 mg/kg) had an increase from baseline in QT duration corrected for heart rate by Fridericia's formula and by Bazett's formula >60 msec at 4 h postdose. Oxygen saturation was consistent across dose levels, and there were no notable changes or trends observed (data not shown). There was no evidence of immunogenicity defined by the presence of anti‐ACE2 binding antibodies (data not shown).

**Table 3:** Summary of all adverse events by GSK2586881 cohort

Event (*n*)	Cohort 10.1 mg/kg (*n* = 4)	Cohort 20.2 mg/kg (*n* = 5)	Cohort 30.4 mg/kg (*n* = 6)	Cohort 40.8 mg/kg (*n* = 8)	Total (*N* = 23)
Any event	1	3	5	2	11
Back pain	1	0	2	0	3
Pain in extremity	0	0	1	0	1
Pneumonia	0	0	1	0	1
Upper respiratory tract infection	0	1	0	0	1
Viral upper respiratory tract infection	0	0	1	0	1
Bradycardia	0	0	0	1	1
Headache^a^	0	0	0	1	1
Cough	0	1	0	0	1
Erythema^a^	0	1	0	0	1

^a^ Recorded as drug‐related.

### Systemic and pulmonary wedge RAS peptides and renin

**Figure 4:**
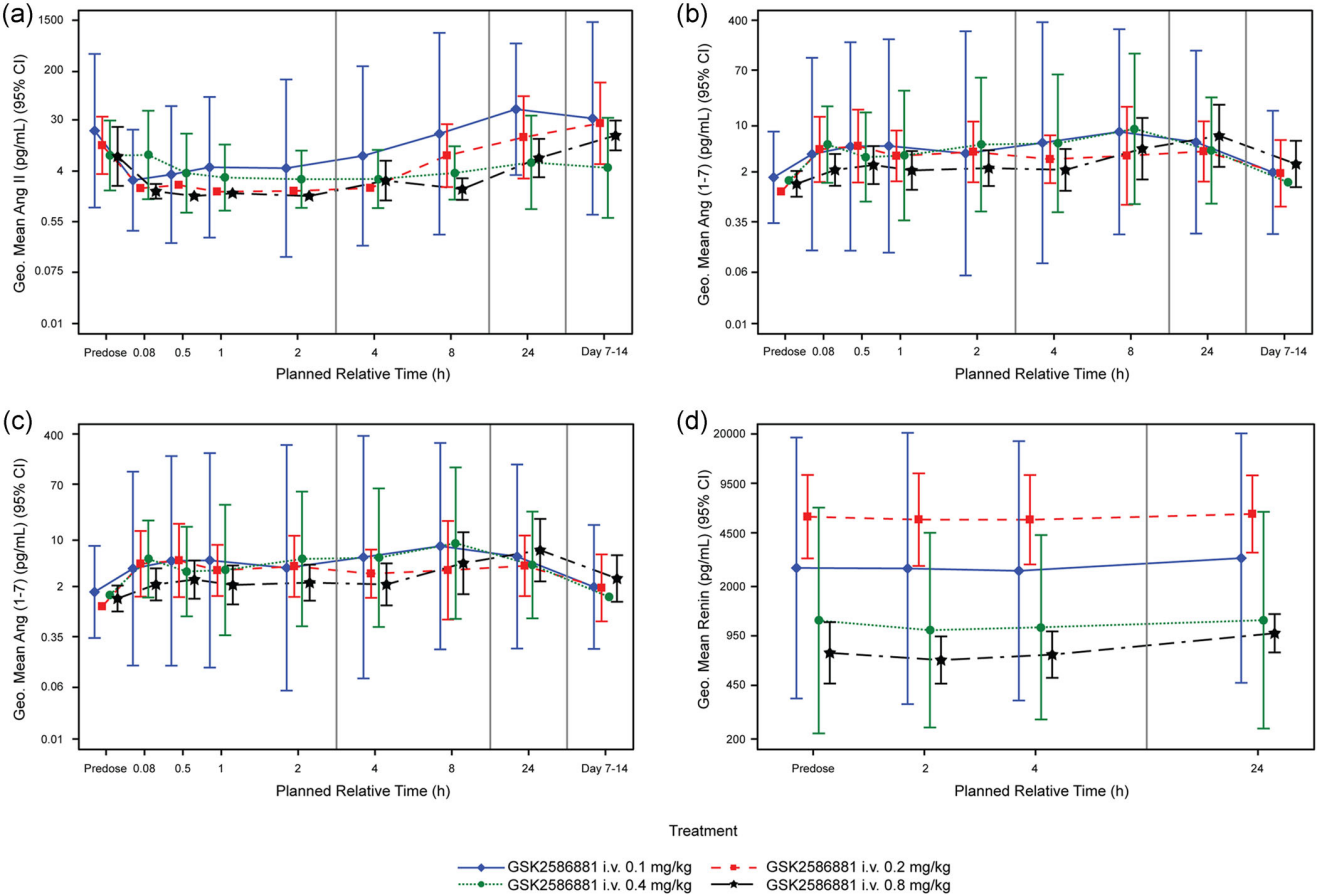
Systemic renin‐angiotensin system peptide concentrations before and following treatment with GSK2586881. Geometric mean time profiles and 95% confidence intervals (denoted as 95% CI; evaluable population) for (a) Ang II, (b) Ang(1–7), (c) Ang II/Ang(1–7) ratio, and (d) renin. Ang, angiotensin; i.v., intravenous

Ang II was present at quantifiable concentrations in predose samples in 19/22 participants before GSK2586881 treatment. Treatment with GSK2586881 at all doses resulted in a reduction in the peripheral venous concentration of Ang II in all participants who had detectable levels of Ang II before treatment; variation was seen in the time course and magnitude of this reduction. There was a trend for the duration of the reduction to be longer in participants who received higher doses of GSK2586881 (Figure 4a). The concentration of Ang II increased again after 4 h in all cohorts except Cohort 4 (0.8 mg/kg), in which it increased by 24 h. Ang II concentration showed evidence of returning to predose levels in the 24 h postdose and follow‐up samples (7–14 days postdose), although there was some variability. Some participants had elevated levels of Ang II at these time‐points and others showed a sustained reduction. The concentration of Ang(1–7), the product of GSK2586881 acting on Ang II, was low or undetectable in the venous blood of all participants at baseline but increased rapidly to detectable levels in the majority of participants (15/22) after treatment with GSK2586881 (Figure 4b). These elevations were sustained for at least 24 h in most participants but showed evidence of returning towards predose levels in the follow‐up samples (7–14 days postdose). As seen with Ang(1–7), levels of Ang(1–5), a degradation product of Ang(1–7), were low to undetectable before treatment with GSK2586881, but rapidly increased after dosing to levels that were maintained for at least 24 h (data not shown). The ratio of the concentration of Ang II to Ang(1–7), a more sensitive measure of the activity of ACE2 than measurement of these peptides individually, showed the expected clear decrease after treatment with GSK2586881 (Figure 4c). All doses of GSK2586881 tested were supramaximally effective at decreasing Ang II/Ang(1–7) ratio, hence no concentration‐dependent response was observed in the magnitude of the decrease in Ang II/Ang(1–7) ratio. However, there was a trend towards a longer duration of reduction in Ang II/Ang(1–7) ratio at higher doses of GSK2586881. There was a good correlation between RAS peptide levels in the venous and wedge RAS blood samples. While consistent among samples from the same participant, circulating renin concentration was extremely variable between participants in this study. There was a weak tendency for Ang II levels in predose and 24 h postdose samples to be higher in subjects with higher renin concentrations and a similar trend for Ang(1–7) and renin in 2 and 4 h postdose samples (data not shown). However, there was no consistent impact of treatment with GSK2586881 on circulating renin concentration, the key rate‐determining enzyme in the RAS, in any participant at any dose or any time‐point after administration (Figure 4d).

### Disease activity biomarkers (NT‐proBNP and NO_*x*_^−^)

Consistent with the observed lack of impact of GSK2586881 on cardiopulmonary hemodynamic parameters, there was limited impact of GSK2586881 on circulating levels of NT‐proBNP, or on the concentration of total NO_*x*_^−^ or the individual species (nitrite and nitrate), at any dose or time‐point following treatment up to 24 h postdose (data not shown).

### Plasma concentrations and PK of GSK2586881

**Figure 5:**
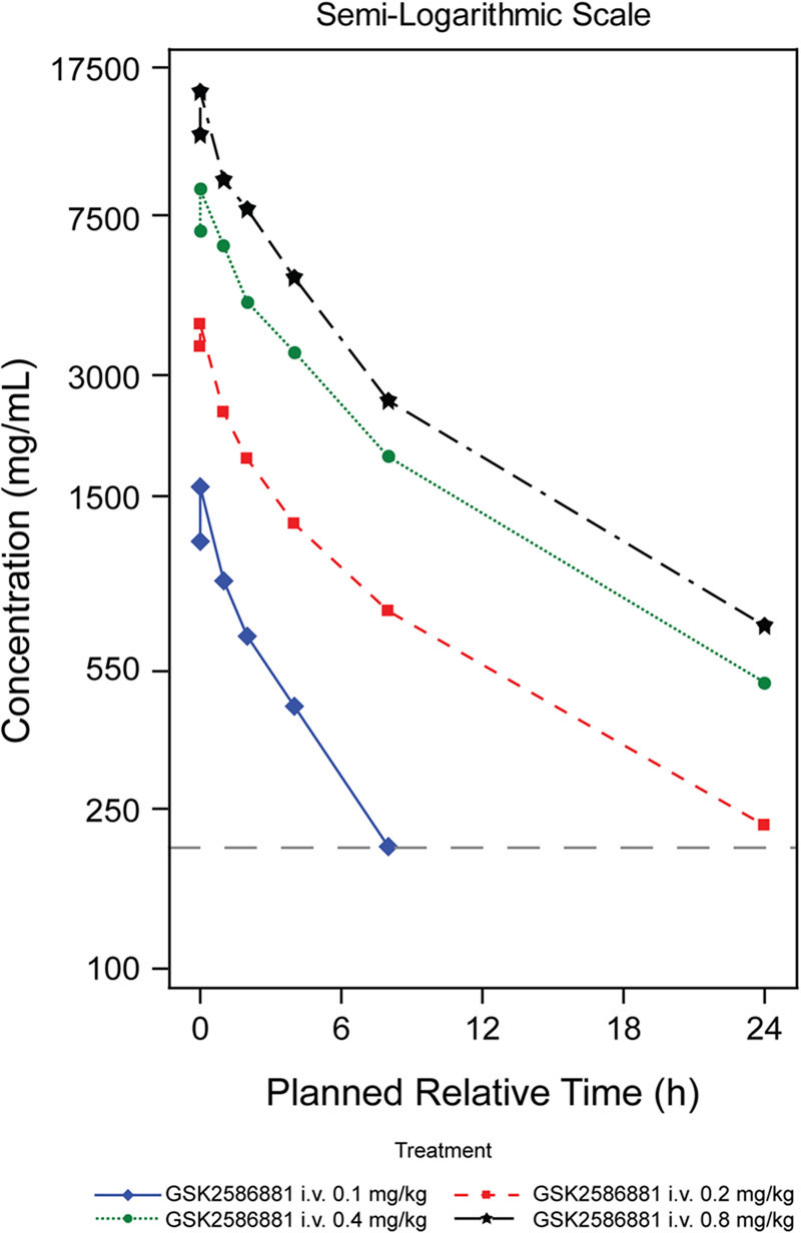
Mean GSK2586881 plasma concentrations. The dashed line represents the lower limit of quantification (200 ng/ml). i.v., intravenous

For the majority of participants, GSK2586881 plasma concentrations were quantifiable (>200 ng/ml) up to 24 h poststart of infusion (0.2–0.8 mg/kg) and up to 8 h poststart of infusion at the lowest dose investigated (0.1 mg/kg). In most participants, maximum concentrations (C_max_) were observed in samples collected at the first sampling time poststart of infusion (Figure 5), with the exception of four participants where C_max_ was reached at 0.5 h poststart of infusion (the second sampling time poststart of infusion). Following IV infusion, plasma concentrations of GSK2586881 declined rapidly from peak in a bi‐phasic manner, with an apparent terminal half‐life of approximately 7 h. The PK profiles for GSK2586881 were as expected and while formal statistical assessment of dose proportionality was not performed, fold increases and visual inspection of the 95% confidence intervals for the geometric means for C_max_ and area under the plasma concentration‐time curve suggested a reasonable approximation of dose proportionality over the dose range of 0.1–0.8 mg/kg.

### PAH type and effect of GSK2586881

Individual patient data showing the effect of GSK2586881 on Ang II/Ang(1–7) ratio, and on mPAP, CI, and PVR by dose and PAH type (idiopathic, heritable, associated with collagen vascular disease) are presented in Figure S1 and Table S4. Overall, there is no correlation between the type of PAH and response to GSK2586881, in terms of cardiopulmonary hemodynamics or Ang II/Ang(1–7) ratio.

### DISCUSSION

#### Cardiopulmonary hemodynamics

The primary objective of this exploratory dose‐escalation study was to determine if single IV doses of GSK2586881 (0.1–0.8 mg/kg) could impact acute cardiopulmonary hemodynamics in PAH patients receiving standard of care PAH therapy (including background therapy). This study was based on a single‐center pilot study, which suggested that single doses of GSK2586881 might acutely vasodilate the pulmonary vasculature. In the pilot study, five PAH patients on background therapy were administered a single IV dose of GSK2586881 (three at 0.2 mg/kg and two at 0.4 mg/kg), which demonstrated a significant trend for an increase in cardiac output and a reduction in PVR (between 1 and 4 h postdose).^23^

Increased RAS pathway activation is associated with pulmonary vascular dysfunction. Ang II has a promitogenic role in pulmonary vascular smooth muscle cells^25^, ^26^ and increased Ang II levels at baseline are associated with disease progression in patients with idiopathic PAH,^11^ demonstrating that increased RAS pathway activation is linked to pulmonary vascular dysfunction/remodeling. In an animal model of PAH, the ACE2/Ang(1–7)/Mas axis exerted cardiopulmonary protective effects, suggesting that ACE2/Ang(1–7) overexpression could have a role in inhibiting vascular remodeling in PAH.^27^ There is, therefore, a rationale for modulation of the RAS via exogenous supplementation of ACE2 to rebalance Ang II/Ang(1–7) levels in the treatment of PAH. Strategies to boost the expression and/or activity of ACE2 have demonstrated significant antiremodeling efficacy in various animal models of PAH.^28^, ^29^, ^30^ Direct administration of GSK2586881 exhibits antiremodeling effects in animal models of pulmonary hypertension, with significant attenuation of pulmonary vascular remodeling observed in the murine bleomycin model^31^ and reversal of established pulmonary hypertension in a mouse model of familial PAH.^18^ GSK2586881 has also been shown to directly suppress pathological remodeling of the right ventricle, leading to enhanced cardiac performance in a murine right ventricular pressure overload model.^17^ Previous clinical studies of GSK2586881 in mechanically ventilated patients with acute respiratory distress syndrome^22^ and patients with PAH^23^ demonstrated rapid modulation of RAS peptides, indicating a potential role for GSK2586881 in modulation of the RAS pathway as a means to lessen pulmonary vascular dysfunction.

In the present study, single IV doses of GSK2586881 did not lead to meaningful postdose changes in mean hemodynamic parameters (PVR, CI, and mPAP), so the primary endpoint of the study was not achieved. There were also no significant changes in other hemodynamic measures. Additional analysis of background PAH therapy in individual participants demonstrated no correlation between the degree of background vasodilator therapy and response. No differences in results were seen when stratified by the etiology of PAH.

Of note, this study used an additional higher dose than the pilot study yet was still not able to detect meaningful hemodynamic changes. The difference in results between this study and the pilot study may be due to differences in study design, sample size, participant characteristics, and/or data presentation. The sample size of the pilot study (*N* = 5) was smaller than that of the present study (*N* = 23), which may have limited the generalizability of the results. With respect to the study population, all patients in the pilot study had idiopathic PAH, whereas this study also included patients with heritable and connective tissue disease‐associated PAH, who together comprised about half the cohort; however, no differences in results were seen when stratified by etiology of PAH. There may have been differences in baseline PAH treatment between this study and the pilot study; however, almost all patients in both studies were on at least dual combination therapy. Mean hemodynamics, including PVR, CI, and mPAP, were comparable at baseline between the pilot study and the present study (within ±2 Wood units, ±1 L/min/m^2^, and ±6 mmHg, respectively).

Any treatment effect was hypothesized to be rapid in onset due to the enzymatic mechanism of action of GSK2586881, so 1 h postdose was determined to be the key time‐point of interest. However, a consistent and sustained response was important, and this was expected given the PK properties of GSK2586881 and the kinetics of RAS peptide changes. The response criteria were not achieved at any dose level or time‐point for mean change in PVR and CI.

The reasons for the discrepancy between the positive preclinical data and the lack of response in the current study are not fully understood. Possible explanations include underdosing, not reaching sufficient local concentrations, insufficient target modulation, and the complexity of the RAS in PAH patients. Nevertheless, whether repetitive doses of GSK2586881 over a longer period of time would lead to changes in pulmonary vascular tone or remodeling remains unknown.

#### Safety and tolerability, biomarkers, and PK

GSK2586881 was well tolerated when administered as a single IV dose at 0.1, 0.2, 0.4, or 0.8 mg/kg to participants with PAH in this study, with a safety profile consistent with completed clinical studies to date.^21^, ^22^ ECG evaluation demonstrated that GSK2586881 did not affect the cardiac conduction system. Following the infusion, Ang II concentrations reduced rapidly, often to below the limit of quantification. These reductions were typically evident by the end of the infusion period. The data were consistent with the expected reversible reduction of Ang II concentration after treatment with an enzyme that degrades this peptide, confirming infused ACE2 was active at the target. The concentrations of Ang(1–7) and Ang(1–5) showed sustained increases following infusion of GSK2586881. This result was expected, as Ang II is cleaved by ACE2 to Ang(1–7), which is subsequently metabolized by ACE to Ang(1–5).^14^, ^15^, ^16^ There was a trend for the duration of the reduction in Ang II concentration to be sustained for longer at higher doses of GSK2586881. On average, the elevations in Ang(1–7) and Ang(1–5) were maintained for at least 24 h. By the follow‐up visit, concentrations had begun to return to pretreatment levels. These results were as expected and confirm the pharmacological activity of GSK2586881 in vivo. There was no clear relationship between Ang II concentration, Ang(1–7) concentration, or Ang II/Ang(1–7) ratio and cardiopulmonary hemodynamic measurements, suggesting that Ang II does not play a key role in mediating the acute vasodilatory response in World Health Organization functional class I PAH. This result is in contrast to the pilot study, in which a single IV dose of GSK2586881 increased the Ang II/Ang(1–7) ratio, with a concomitant increase in cardiac output.^23^ These contrasting results may be due to differences in the study designs.

There was no effect of GSK2586881 treatment on the circulating concentration of renin in this study. This, along with the very high variability in measured renin concentrations (and indeed Ang II concentrations) in this study population, may indicate dysregulation of the RAS in PAH patients. It is unclear whether this is a consequence of the disease or the impact of ongoing therapy. ACE activity is high in the pulmonary circulation; the lungs are thought to be a major source of circulating Ang II.^32^, ^33^ Therefore, the relationship between RAS peptide levels measured in peripheral venous blood samples and those in blood taken from the wedge position was evaluated to determine whether the concentration of Ang II in the periphery reflects the concentration entering the lung. Overall, there was a good correlation between the venous and wedge RAS blood samples, demonstrating that RAS peptide concentrations in peripheral blood samples provide a reliable measure of RAS peptide concentrations in blood entering the lung.

GSK2586881 plasma concentrations demonstrated that participants were rapidly exposed, and systemic exposure was maintained over the period of the cardiopulmonary hemodynamics assessments (4 h). Systemic exposure appeared to increase in an approximately dose‐proportional manner over the dose range of 0.1 to 0.8 mg/kg. The PK characteristics of GSK2586881 in patients with PAH were consistent with historical data in healthy participants.^21^

NT‐proBNP is a biomarker of cardiac stress or ventricular workload and—in the context of PAH—decreases as a result of the reduced force of contraction if pulmonary blood pressure/right ventricular afterload is reduced. The vasodilatory effects of Ang(1–7) are thought to be due to the release of nitric oxide from the vascular endothelium. Nitric oxide can be converted into the more stable species nitrite and nitrate in vivo and these analytes are commonly used as biomarkers of nitric oxide, although they may not be sensitive to local changes in this mediator. There was no meaningful impact of treatment with GSK2586881 at any dose on circulating concentrations of NO_*x*_^−^ or NT‐proBNP up to 24 h after dosing, indicating no acute effects on these biomarkers, which was consistent with the lack of hemodynamic effect observed.

#### Limitations

The ability to observe an impact of GSK2586881 on acute cardiopulmonary hemodynamics may have been hampered by recruiting PAH patients actively treated with vasodilators as background therapy. However, recruiting only treatment‐naïve patients for this study would have been challenging. This was an open‐label study of a single dose per cohort. An open‐label study design may introduce bias; however, the bias would typically favor observing a false‐positive response. Furthermore, it was not possible to determine the expected level of change from baseline in the key cardiopulmonary endpoints due to the lack of a placebo comparator. Responses to chronic administration were not studied. This is particularly important given the reported variability in assessing cardiopulmonary hemodynamics using cardiac catheterization.^34^ As dysregulation of the RAS pathway has been suggested to underlie pulmonary vascular remodeling in PAH,^11^, ^12^ it is conceivable that repeat dosing of GSK2586881 for a sufficient duration would be required to elicit an impact on cardiopulmonary hemodynamics. This single‐dose study was not designed to examine the potential impact of GSK2586881 on vascular remodeling.

## CONCLUSIONS

A single IV dose of GSK2586881 (0.1, 0.2, 0.4, and 0.8 mg/kg) was well tolerated in participants with PAH, with no SAEs and no evidence of immunogenicity observed. GSK2586881 was quantifiable in plasma for up to 4 h poststart of infusion in all participants, but our results suggest that GSK2586881 had no consistent or sustained effect on acute cardiopulmonary hemodynamics in participants with PAH receiving background PAH therapy. GSK2586881 caused a consistent and sustained reduction in Ang II and a corresponding increase in Ang(1–7) and Ang(1–5), confirming the pharmacological activity of GSK2586881 in vivo. While there does not appear to be a consistent acute vasodilatory response to single doses of GSK2586881 in participants with PAH, the potential benefits in terms of chronic vascular remodeling remain to be determined.

## CONFLICT OF INTERESTS

Marc A. Simon: Consultancy for Acceleron, Actelion, Altavant Sciences, Bial—Portela C S.A., and United Therapeutics. Research grants from Novartis. Hemodynamic core lab work for Aadi. Supported by NIH grants R01AG058659 and P01HL103455. Fernando Torres: Personal fees from Actelion, Bayer, Reata, and Arena and grants from Gilead, United Therapeutics, Medtronic, Eiger, and Bellerophon. Ekkehard Grünig: Research grants and lecture fees from Actelion/Janssen and Bayer/MSD, research grants from GSK, United Therapeutics, and Novartis, and lecture fees from SCOPE, OrPha Swiss GmbH, and Zurich Heart House. Pilar Escribano‐Subias: Consultancy and/or lectures for Acceleron, Actelion, Janssen, MSD United Therapeutics, Ferrer, and Abbott. Research grants to institutions from Janssen and Ferrer. Manuel López Meseguer: Remunerations for consultancy and/or lectures from Janssen, MSD, and GSK. Michael Halank: Wages for consultancy and/or lectures from Acceleron, Actelion, AstraZenca, Bayer, BerlinChemie, GSK, Janssen, MSD, and Novartis. Christian Opitz: Institution has received speaker fees and honoraria for consultations from Actelion, Bayer, GlaxoSmithKline, Novartis, and Pfizer. Stephan Rosenkranz: Remunerations for consultancy and/or lectures from Abbott, Acceleron, Actelion, AstraZeneca, Bayer, BMS, Janssen, MSD, Novartis, Pfizer, United Therapeutics. Research grants to institution from Actelion, AstraZeneca, Bayer, Novartis; Deutsche Forschungsgemeinschaft (DFG), Bundesministerium für Bildung und Forschung (BMBF), and Else‐Kröner‐Fresenius‐Stiftung (EKFS). Kate Hanrott, David A. Hall, Deborah Hewens, William M. Powley, David C. Budd, Sarah Siederer, Aili L. Lazaar, and Anthony Cahn are employees and stock/shareholders of GlaxoSmithKline plc.

## ETHICAL APPROVAL

All participants provided written informed consent for participation in this study.

## AUTHOR CONTRIBUTIONS

Kate Hanrott, David A. Hall, David C. Budd, Deborah Hewens, William M. Powley, Sarah Siederer, Aili L. Lazaar, Anthony Cahn, and Andrew Bayliffe were involved in study conception/design; Marc A. Simon, Stephan Rosenkranz, Pilar Escribano‐Subias, Manuel López Meseguer, Christian Opitz, Michael Halank, Ekkehard Grünig, and Fernando Torres were involved in data acquisition; all authors were involved in data analysis and/or interpretation. All authors were involved in writing/critical review of draft versions of this manuscript and all approved the final version for submission for publication.

## Supporting information

Supporting information.

Supporting information.
