# Supplementary material for: An open‐label, dose‐escalation study to evaluate the safety, tolerability, pharmacokinetics, and pharmacodynamics of single doses of GSK2586881 in participants with pulmonary arterial hypertension
Source: Pulm Circ. 2022 Jan 20;12(1):e12024. doi: 10.1002/pul2.12024 (PMC9053011; doi:10.1002/pul2.12024)
Supplement: Supplementary file 1 — Supporting information. [file PUL2-12-e12024-s002.docx]

**SUPPLEMENT**

**Supplementary methods**

The final planned primary analyses were performed after all participants completed the study as defined in the protocol.

The primary analyses for pulmonary hemodynamic endpoints were based on the evaluable population. For each endpoint, a Bayesian analysis of the change from baseline was conducted, adjusting for time-point, dose, baseline, dose by time-point, and baseline by time-point. Non-informative priors were assumed. Data for each endpoint were log transformed prior to analysis, with change from baseline geometric means at each post-dose time-point representing a percentage increase/decrease from baseline (presented as a ratio to baseline).

Posterior distributions were used to assess probability statements for changes from baseline of interest: pulmonary vascular resistance: posterior probability of any percentage decrease from baseline (ratio <1), 15% (ratio <0.85), 20% (ratio <0.80), 25% (ratio <0.75); and cardiac index: posterior probability of any percentage increase from baseline (ratio >1), 5% (ratio >1.05), 6% (ratio >1.06), 7.5% (ratio >1.075). These values are based on previous literature demonstrating a positive impact on hemodynamics following a single dose of vasodilators in patients with PAH.^1, 2^

**Supplementary Table 1. Schedule of assessments.**

| **Procedure** | **Screening  (up to 28  days before  dosing)** | **Treatment Period** | | | | | | | | | **Follow- up**  **Day 7–14** | **Follow- up**  **Day 28 ± 3 days** | **Notes** |
| --- | --- | --- | --- | --- | --- | --- | --- | --- | --- | --- | --- | --- | --- |
|  |  | **Pre-  dose^*^** | **0 h** | **0.08 h (5min)^†^** | **0.5 h** | **1 h** | **2 h** | **4 h** | **8 h** | **24 h** |  |  |  |
| Informed consent | X |  |  |  |  |  |  |  |  |  |  |  |  |
| Genetics consent | X |  |  |  |  |  |  |  |  |  |  |  | Genetics consent was optional |
| Inclusion and exclusion criteria | X |  |  |  |  |  |  |  |  |  |  |  | Clinical status rechecked before randomization and/or first dose of study medication |
| Demography | X |  |  |  |  |  |  |  |  |  |  |  |  |
| Full physical examination including height and weight | X |  |  |  |  |  |  |  |  |  |  |  |  |
| Medical history (includes substance usage and family history of premature CV disease) | X |  |  |  |  |  |  |  |  |  |  |  | Substances: drugs, alcohol, tobacco and caffeine |
| Past and current medical conditions | X |  |  |  |  |  |  |  |  |  |  |  |  |
| Serum OR urine pregnancy test (WOCBP only) | X | X |  |  |  |  |  |  |  |  | X |  |  |
| FSH and estradiol test (postmenopausal females only as needed) | X |  |  |  |  |  |  |  |  |  |  |  |  |
| HIV, hepatitis B and C screening | X |  |  |  |  |  |  |  |  |  |  |  | If test performed within 3 months prior to first dose of study treatment, testing at screening was not required |
| Functional classification | X |  |  |  |  |  |  |  |  |  |  |  |  |
| 6-minute walk distance | X^‡^ |  |  |  |  |  |  |  |  |  |  |  |  |
| Admission |  | X |  |  |  |  |  |  |  |  |  |  | Participants were admitted the day before dosing to enable completion of required pre-dose time-point assessments |
| Brief physical |  | X |  |  |  |  |  |  |  |  | X |  |  |
| Study treatment |  |  | X |  |  |  |  |  |  |  |  |  |  |
| Vital signs | X | X |  |  | X | X | X | X | X | X | X |  | Vital signs were measured after 5 minutes supine at all time-points.  Triplicate blood pressure (BP) was taken at screening only |
| Pulse oximetry (SpO_2_) | X | X |  |  | X | X | X | X | X | X | X |  | Pulse oximetry was measured and recorded with each blood pressure assessment |
| Laboratory assessments (including liver chemistries, hematology panel, and coagulation panel) | X | X |  |  |  |  |  |  |  | X | X |  |  |
| Urinalysis | X |  |  |  |  |  |  |  |  | X |  |  |  |
| 12-lead ECG | X | X |  |  |  |  |  | X |  | X | X |  | Triplicate was performed at Screening and Pre-dose |
| Telemetry |  | 🡨=====================================🡪 | | | | | | | | |  |  | Monitoring started 30min prior to treatment administration and continued throughout the study until 24h after dosing |
| Right heart catheter  insertion |  | 🡨===========================🡪 | | | | | | |  |  |  |  | Right heart catheter inserted prior to dose and removed after the 4h hemodynamic measurement |
| Blood sample for biomarkers of disease activity |  | X |  |  |  |  | X | X |  | X |  |  |  |
| Blood sample for nitric oxide |  | X |  |  |  |  | X | X |  | X |  |  |  |
| Blood sample for PK |  | X |  | X | X | X | X | X | X | X |  |  |  |
| Blood sample for immunogenicity |  | X |  |  |  |  |  |  |  |  | X | X | Additional visits were required |
| Blood sample for RAS biomarkers |  | X |  | X | X | X | X | X | X | X | X |  |  |
| Blood sample for pulmonary wedge RAS biomarkers |  | X |  |  |  | X | X | X |  |  |  |  |  |
| Hemodynamic measurements |  | X |  |  |  | X | X | X |  |  |  |  |  |
| Genetic sample |  | X |  |  |  |  |  |  |  |  |  |  | Was taken any time after consent has been signed. Only required once and was optional |
| Discharge |  |  |  |  |  |  |  |  |  | X |  |  |  |
| AE review |  | 🡨========================================🡪 | | | | | | | | | X |  |  |
| SAE review | X | 🡨========================================🡪 | | | | | | | | | X |  |  |
| Concomitant medication review^§^ | X | X |  |  |  |  |  |  |  |  | X |  |  |

^*^Re-dose measurements were taken any time after admission up until dosing; ^†^Procedures were completed immediately after dosing was completed; ^‡^If the six-minute walk test was performed in the last 6 months, and the participant was stable on current medications then there was no need to repeat. Historical data were databased; ^§^Concomitant medications for the 30 days prior (8 weeks prior for PAH medications) were reviewed/recorded at screening to evaluate eligibility and changes were recorded throughout the study.

AE, adverse event; CV, cardiovascular; ECG, electrocardiogram; FSH, follicle-stimulating hormone; HIV, human immunodeficiency virus; PAH, pulmonary arterial hypertension; PK, pharmacokinetics; RAS, renin-angiotensin system; SAE, serious adverse event; WOBCP, women of childbearing potential.

**Supplementary Table 2. Concomitant medications**

| **n (%)** | **Cohort 1**  **GSK2586881 0.1 mg/kg (n=4)** | **Cohort 2 GSK2586881 0.2 mg/kg (n=5)** | **Cohort 3 GSK2586881 0.4 mg/kg (n=6)** | **Cohort 4 GSK2586881 0.8 mg/kg (n=8)** | **Total**  **(N=23)** |
| --- | --- | --- | --- | --- | --- |
| **Cardiovascular system (any medication)** | 4 (100) | 5 (100) | 6 (100) | 7 (88) | 22 (96) |
| MACITENTAN | 2 (50) | 2 (40) | 4 (67) | 3 (38) | 11 (48) |
| AMBRISENTAN | 2 (50) | 2 (40) | 0 | 4 (50) | 8 (35) |
| TADALAFIL | 3 (75) | 2 (40) | 0 | 2 (25) | 7 (30) |
| TORASEMIDE | 1 (25) | 1 (20) | 2 (33) | 2 (25) | 6 (26) |
| ALDACTONE | 0 | 1 (20) | 0 | 3 (38) | 4 (17) |
| FUROSEMIDE | 0 | 2 (40) | 1 (17) | 0 | 4 (17) |
| RIOCIGUAT | 0 | 0 | 2 (33) | 1 (13) | 3 (13) |
| SIMVASTATIN | 0 | 2 (40) | 0 | 1 (13) | 3 (13) |
| SPIRONOLACTONE | 1 (25) | 1 (20) | 1 (17) | 0 | 3 (13) |
| LISINOPRIL | 0 | 0 | 2 (33) | 0 | 2 (9) |
| BISOPROLOL | 0 | 0 | 0 | 1 (13) | 1 (4) |
| BOSENTAN | 0 | 0 | 1 (17) | 0 | 1 (4) |
| CANDESARTAN CILEXETIL | 0 | 0 | 0 | 1 (13) | 1 (4) |
| FISH OIL | 0 | 1 (20) | 0 | 0 | 1 (4) |
| HYDRALAZINE | 0 | 0 | 1 (17) | 0 | 1 (4) |
| HYDROCHLOROTHIAZIDE | 1 (25) | 0 | 0 | 0 | 1 (4) |
| ILOPROST | 0 | 0 | 1 (17) | 0 | 1 (4) |
| OLMESARTAN MEDOXOMIL | 0 | 0 | 1 (17) | 0 | 1 (4) |
| VERAPAMIL | 0 | 1 (20) | 0 | 0 | 1 (4) |
| VERAPAMIL HYDROCHLORIDE | 0 | 0 | 1 (17) | 0 | 1 (4) |
| **Blood and Blood Forming Organs** |  |  |  |  |  |
| RIVAROXABAN | 2 (50) | 0 | 1 (17) | 2 (25) | 4 (17) |
| ACENOCOUMAROL | 2 (50) | 0 | 2 (33) | 0 | 4 (17) |
| PHENPROCOUMON | 0 | 0 | 1 (17) | 3 (38) | 4 (17) |
| SELEXIPAG | 0 | 1 (20) | 1 (17) | 2 (25) | 4 (17) |
| TREPROSTINIL | 2 (50) | 1 (20) | 1 (17) | 0 | 4 (17) |
| EPOPROSTENOL SODIUM | 1 (25) | 2 (40) | 0 | 0 | 3 (13) |
| IRON | 0 | 0 | 2 (33) | 0 | 2 (9) |
| APIXABAN | 1 (25) | 0 | 0 | 0 | 1 (4) |
| ENOXAPARIN SODIUM | 0 | 0 | 0 | 1 (13) | 1 (4) |
| FERINJECT | 0 | 1 (20) | 0 | 0 | 1 (4) |
| FERROUS GLUCONATE | 0 | 1 (20) | 0 | 0 | 1 (4) |
| FERROUS SULPHATE | 0 | 0 | 1 (17) | 0 | 1 (4) |
| SODIUM CHLORIDE | 0 | 0 | 1 (17) | 0 | 1 (4) |
| WARFARIN SODIUM | 0 | 1 (20) | 0 | 0 | 1 (4) |
| **Nervous system** |  |  |  |  |  |
| MEPIVACAINE HYDROCHLORIDE | 0 | 0 | 0 | 4 (50) | 4 (17) |
| PARACETAMOL | 0 | 1 (20) | 3 (50) | 0 | 4 (17) |
| GABAPENTIN | 0 | 1 (20) | 1 (17) | 1 (13) | 3 (13) |
| OXYCODONE | 0 | 2 (40) | 0 | 0 | 2 (9) |
| PIRITRAMIDE | 1 (25) | 0 | 1 (17) | 0 | 2 (9) |
| ACETYLSALICYLIC ACID | 0 | 1 (20) | 0 | 0 | 1 (4) |
| CITALOPRAM | 1 (25) | 0 | 0 | 0 | 1 (4) |
| CYCLOBENZAPRINE HYDROCHLORIDE | 0 | 0 | 1 (17) | 0 | 1 (4) |
| DULOXETINE HYDROCHLORIDE | 0 | 0 | 1 (17) | 0 | 1 (4) |
| ESCITALOPRAM | 0 | 1 (20) | 0 | 0 | 1 (4) |
| HYDROCODONE | 0 | 1 (20) | 0 | 0 | 1 (4) |
| Multiple Ingredients | 0 | 0 | 1 (17) | 0 | 1 (4) |
| SERTRALINE | 1 (25) | 0 | 0 | 0 | 1 (4) |
| TRAMADOL HYDROCHLORIDE | 0 | 1 (20) | 0 | 0 | 1 (4) |
| TRAZODONE | 0 | 1 (20) | 0 | 0 | 1 (4) |
| **Alimentary tract and metabolism** |  |  |  |  |  |
| OMEPRAZOLE | 2 (50) | 1 (20) | 1 (17) | 1 (13) | 5 (22) |
| COLECALCIFEROL | 0 | 2 (40) | 1 (1) | 1 (13) | 4 (17) |
| PANTOPRAZOLE | 0 | 1 (20) | 1 (17) | 1 (13) | 3 (13) |
| POTASSIUM CHLORIDE | 1 (25) | 1 (20) | 1 (17) | 0 | 3 (13) |
| ASCORBIC ACID | 0 | 0 | 2 (33) | 0 | 2 (9) |
| BUDESONIDE | 0 | 0 | 1 (17) | 0 | 1 (4) |
| CULTURELLE | 0 | 1 (20) | 0 | 0 | 1 (4) |
| DAPAGLIFLOZIN PROPANEDIOL | 0 | 0 | 1 (17) | 0 | 1 (4) |
| DOMPERIDONE | 1 (25) | 0 | 0 | 0 | 1 (4) |
| ERGOCALCIFEROL | 0 | 1 (20) | 0 | 0 | 1 (4) |
| LOPERAMIDE HYDROCHLORIDE | 0 | 1 (20) | 0 | 0 | 1 (4) |
| METFORMIN | 0 | 0 | 1 (17) | 0 | 1 (4) |
| ONDANSETRON | 0 | 1 (20) | 0 | 0 | 1 (4) |
| PREDNISONE | 0 | 0 | 1 (17) | 0 | 1 (4) |
| PROTONIX | 0 | 0 | 1 (17) | 0 | 1 (4) |
| VITAMIN D | 0 | 0 | 1 (17) | 0 | 1 (4) |
| VITAMINS | 0 | 1 (20) | 0 | 0 | 1 (4) |
| **Genito urinary system and sex hormones** |  |  |  |  |  |
| SILDENAFIL | 1 (25) | 2 (4) | 2 (33) | 0 | 5 (22) |
| SILDENAFIL CITRATE | 0 | 1 (20) | 1 (17) | 3 (38) | 5 (22) |
| Multiple Ingredient | 0 | 1 (20) | 0 | 1 (13) | 2 (9) |
| TADALAFIL | 0 | 0 | 0 | 1 (13) | 1 (4) |
| **Respiratory system** |  |  |  |  |  |
| Multiple Ingredients | 0 | 2 (40) | 2 (33) | 1 (13) | 5 (22) |
| OXYGEN | 0 | 2 (40) | 1 (17) | 1 (13) | 4 (17) |
| CETIRIZINE | 0 | 0 | 0 | 2 (25) | 2 (9) |
| SALBUTAMOL | 0 | 0 | 1 (17) | 1 (13) | 2 (9) |
| AFRIN | 0 | 1 (20) | 0 | 0 | 1 (4) |
| BENZONATATE | 0 | 1 (20) | 0 | 0 | 1 (4) |
| FENOTEROL | 0 | 0 | 1 (17) | 0 | 1 (4) |
| FEXOFENADINE HYDROCHLORIDE | 0 | 1 (20) | 0 | 0 | 1 (4) |
| GUAIFENESIN | 0 | 1 (20) | 0 | 0 | 1 (4) |
| TIOTOPIUM BROMIDE | 0 | 0 | 1 (17) | 0 | 1 (4) |
| **Musculo-skeletal system** |  |  |  |  |  |
| HYDROXYCHLOROQUINE | 0 | 1 (20) | 2 (33) | 1 (13) | 4 (17) |
| ALLOPURINOL | 0 | 1 (20) | 1 (17) | 0 | 2 (9) |
| DIAZEPAM | 1 (25) | 0 | 0 | 0 | 1 (4) |
| FEBUXOSTAT | 1 (25) | 0 | 0 | 0 | 1 (4) |
| GLUCOSAMINE | 0 | 1 (20) | 0 | 0 | 1 (4) |
| KETOROLAC TROMETAMOL | 0 | 0 | 1 (17) | 0 | 1 (4) |
| MELOXICAM | 0 | 0 | 1 (17) | 0 | 1 (4) |
| **Systemic hormones, excluding sex hormones and insulin** |  |  |  |  |  |
| LEVOTHYROXINE SODIUM | 2 (50) | 1 (20) | 1 (17) | 2 (25) | 6 (26) |
| Multiple Ingredients | 0 | 0 | 0 | 1 (17) | 1 (4) |
| PREDNISONE | 0 | 1 (20) | 0 | 0 | 1 (4) |
| **Systemic anti-infectives** |  |  |  |  |  |
| AMOXICILLIN | 2 (25) | 0 | 1 (17) | 0 | 3 (13) |
| CEFTRIAXONE SODIUM | 0 | 0 | 1 (17) | 0 | 1 (4) |
| DOXYCYCLINE | 0 | 1 (20) | 0 | 0 | 1 (4) |
| **Dermatologicals** |  |  |  |  |  |
| DESONIDE | 0 | 1 (20) | 0 | 0 | 1 (4) |
| FLONASE | 0 | 1 (20) | 0 | 0 | 1 (4) |
| FLUCONAZOLE | 0 | 0 | 1 (17) | 0 | 1 (4) |
| LIDOCAINE | 0 | 0 | 0 | 1 (13) | 1 (4) |
| PETROLATUM | 0 | 1 (20) | 0 | 0 | 1 (4) |
| **Various** |  |  |  |  |  |
| AMBIGUOUS MEDICATION NOS | 0 | 1 (20) | 1 (17) | 0 | 2 (9) |
| IOMEPROL | 0 | 0 | 1 (17) | 0 | 1 (4) |
| **Antineoplastic and immunomodulating agents** |  |  |  |  |  |
| METHOTREXATE SODIUM | 0 | 1 (20) | 0 | 0 | 1 (4) |
| **Sensory organs** |  |  |  |  |  |
| HEPARIN | 0 | 0 | 1 (17) | 0 | 1 (4) |

A medication may be included in more than one category and appear more than once.

**Supplementary Table 3. Repeated measures Bayesian statistical analysis of change from baseline in hemodynamics (evaluable population) by GSK2586881 cohort.**

| **Parameter** | **Posterior probability of ratio** | **Planned relative time** | **0.1 mg/kg**  **(n=4)** | **0.2 mg/kg**  **(n=5)** | **0.4 mg/kg**  **(n=6)** | **0.8 mg/kg**  **(n=8)** |
| --- | --- | --- | --- | --- | --- | --- |
| PVR, Wood units | <0.85 (>15% decrease) | 1 h | 0.221 | 0.327 | 0.682 | 0.019 |
|  |  | 2 h | 0.216 | 0.007 | 0.123 | 0.019 |
|  |  | 4 h | 0.255 | 0.402 | 0.194 | 0.003 |
| CI, L/min/m^2^ | >1.06 (>6% increase) | 1 h | 0.448 | 0.859 | 0.467 | 0.044 |
|  |  | 2 h | 0.299 | 0.689 | 0.006 | 0.087 |
|  |  | 4 h | 0.760 | 0.953 | 0.002 | 0.052 |

Bayesian analysis of change from baseline for each parameter adjusting for time-point, dose, baseline, dose by time-point, and baseline by time-point interactions. Non-informative priors assumed. Data were log transformed prior to analysis, therefore results represent a ratio from baseline to post-dose.

CI, cardiac index; PVR, pulmonary vascular resistance.

**Supplementary Table 4: Effect of single dose GSK2586881 (0.1, 0.2, 0.4, and 0.8 mg/kg) on Ang II/Ang (1-7), mPAP, CI, and PVR in patients with idiopathic, heritable PAH, and PAH associated with collagen vascular disease.**

| **Dose** | **PAH (FC)** | **Dummy Subject No.** | **Endpoint** | **Pre** | **0.08h** | **0.5h** | **1h** | **2h** | **4h** | **8h** | **24h** | **Day 7-14** |
| --- | --- | --- | --- | --- | --- | --- | --- | --- | --- | --- | --- | --- |
| GSK2586881 i.v. 0.1 mg/kg | Idiopathic PAH (II) | 1 | Ang II / Ang (1-7) | 3.21 | 1.50 | 1.50 | 1.50 | 1.50 | 1.50 | 3.36 | 21.20 | 60.70 |
| GSK2586881 i.v. 0.1 mg/kg | Idiopathic PAH (II) | 1 | mPAP (mmHg) | 35.70 |  |  | 29.30 | 28.70 | 30.70 |  |  |  |
| GSK2586881 i.v. 0.1 mg/kg | Idiopathic PAH (II) | 1 | CI (L/min/m2) | 3.20 |  |  | 3.20 | 3.70 | 4.50 |  |  |  |
| GSK2586881 i.v. 0.1 mg/kg | Idiopathic PAH (II) | 1 | PVR (Wood units) | 5.80 |  |  | 4.80 | 4.20 | 3.70 |  |  |  |
| GSK2586881 i.v. 0.1 mg/kg | Idiopathic PAH (II) | 2 | Ang II / Ang (1-7) | 31.06 | 0.22 | 0.35 | 0.41 | 0.56 | 0.62 | 2.34 | 4.75 | 30.73 |
| GSK2586881 i.v. 0.1 mg/kg | Idiopathic PAH (II) | 2 | mPAP (mmHg) | 29.70 |  |  | 32.00 | 31.00 | 29.30 |  |  |  |
| GSK2586881 i.v. 0.1 mg/kg | Idiopathic PAH (II) | 2 | CI (L/min/m2) | 2.80 |  |  | 2.90 | 2.60 | 2.60 |  |  |  |
| GSK2586881 i.v. 0.1 mg/kg | Idiopathic PAH (II) | 2 | PVR (Wood units) | 4.60 |  |  | 5.30 | 5.40 | 5.10 |  |  |  |
| GSK2586881 i.v. 0.1 mg/kg | Idiopathic PAH (II) | 3 | Ang II / Ang (1-7) | 30.10 | 0.66 | 0.34 | 0.84 | 1.50 | 2.11 | 3.82 | 10.06 | 24.20 |
| GSK2586881 i.v. 0.1 mg/kg | Idiopathic PAH (II) | 3 | mPAP (mmHg) | 40.70 |  |  | 36.70 | 40.70 | 39.70 |  |  |  |
| GSK2586881 i.v. 0.1 mg/kg | Idiopathic PAH (II) | 3 | CI (L/min/m2) | 2.70 |  |  | 3.30 | 3.10 | 3.30 |  |  |  |
| GSK2586881 i.v. 0.1 mg/kg | Idiopathic PAH (II) | 3 | PVR (Wood units) | 7.30 |  |  | 6.00 | 7.20 | 5.60 |  |  |  |
| GSK2586881 i.v. 0.1 mg/kg | Heritable PAH (III) | 4 | Ang II / Ang (1-7) | 6.50 | 1.50 | 1.50 | 1.50 | 1.50 | 1.50 | 0.70 | 4.13 | 1.50 |
| GSK2586881 i.v. 0.1 mg/kg | Heritable PAH (III) | 4 | mPAP (mmHg) | 34.00 |  |  | 34.70 | 31.30 | 39.70 |  |  |  |
| GSK2586881 i.v. 0.1 mg/kg | Heritable PAH (III) | 4 | CI (L/min/m2) | 3.40 |  |  | 3.20 | 3.00 | 3.20 |  |  |  |
| GSK2586881 i.v. 0.1 mg/kg | Heritable PAH (III) | 4 | PVR (Wood units) | 4.90 |  |  | 4.80 | 4.30 | 6.10 |  |  |  |
| GSK2586881 i.v. 0.2 mg/kg | Idiopathic PAH (II) | 5 | Ang II / Ang (1-7) | 27.00 | 0.13 | 0.09 | 0.28 | 0.19 | 0.25 | 1.03 | 4.86 | 45.17 |
| GSK2586881 i.v. 0.2 mg/kg | Idiopathic PAH (II) | 5 | mPAP (mmHg) | 36.30 |  |  | 35.00 | 34.70 | 34.00 |  |  |  |
| GSK2586881 i.v. 0.2 mg/kg | Idiopathic PAH (II) | 5 | CI (L/min/m2) | 3.10 |  |  | 3.80 | 3.30 | 3.40 |  |  |  |
| GSK2586881 i.v. 0.2 mg/kg | Idiopathic PAH (II) | 5 | PVR (Wood units) | 5.30 |  |  | 3.80 | 5.40 | 4.70 |  |  |  |
| GSK2586881 i.v. 0.2 mg/kg | Idiopathic PAH (II) | 6 | Ang II / Ang (1-7) | 24.60 | 1.46 | 2.64 | 0.95 | 1.13 | 1.90 | 7.29 | 4.95 | 14.80 |
| GSK2586881 i.v. 0.2 mg/kg | Idiopathic PAH (II) | 6 | mPAP (mmHg) | 35.00 |  |  | 36.30 | 36.00 | 34.00 |  |  |  |
| GSK2586881 i.v. 0.2 mg/kg | Idiopathic PAH (II) | 6 | CI (L/min/m2) | 4.90 |  |  | 5.40 | 4.70 | 6.80 |  |  |  |
| GSK2586881 i.v. 0.2 mg/kg | Idiopathic PAH (II) | 6 | PVR (Wood units) | 2.90 |  |  | 3.00 | 3.30 | 2.10 |  |  |  |
| GSK2586881 i.v. 0.2 mg/kg | Idiopathic PAH (III) | 7 | Ang II / Ang (1-7) | 6.43 | 0.21 | 0.22 | 0.28 | 0.33 | 0.47 | 1.24 | 3.64 | 5.38 |
| GSK2586881 i.v. 0.2 mg/kg | Idiopathic PAH (III) | 7 | mPAP (mmHg) | 31.30 |  |  | 31.70 | 34.00 | 37.30 |  |  |  |
| GSK2586881 i.v. 0.2 mg/kg | Idiopathic PAH (III) | 7 | CI (L/min/m2) | 2.70 |  |  | 2.50 | 2.60 | 3.10 |  |  |  |
| GSK2586881 i.v. 0.2 mg/kg | Idiopathic PAH (III) | 7 | PVR (Wood units) | 4.60 |  |  | 4.90 | 5.50 | 4.90 |  |  |  |
| GSK2586881 i.v. 0.2 mg/kg | CVD-PAH (II) | 8 | Ang II / Ang (1-7) | 3.16 | 1.50 | 1.50 | 1.50 | 1.50 | 1.50 | 1.50 | 1.50 | 4.42 |
| GSK2586881 i.v. 0.2 mg/kg | CVD-PAH (II) | 8 | mPAP (mmHg) | 32.00 |  |  | 31.30 | 30.70 | 29.30 |  |  |  |
| GSK2586881 i.v. 0.2 mg/kg | CVD-PAH (II) | 8 | CI (L/min/m2) | 4.50 |  |  | 5.00 | 4.80 | 5.10 |  |  |  |
| GSK2586881 i.v. 0.2 mg/kg | CVD-PAH (II) | 8 | PVR (Wood units) | 3.00 |  |  | 2.10 | 2.90 | 2.10 |  |  |  |
| GSK2586881 i.v. 0.2 mg/kg | CVD-PAH (III) | 9 | Ang II / Ang (1-7) | 11.90 | 0.36 | 0.30 | 0.30 | 0.19 | 0.36 | 2.86 | 5.17 | 30.80 |
| GSK2586881 i.v. 0.2 mg/kg | CVD-PAH (III) | 9 | mPAP (mmHg) | 39.30 |  |  | 38.70 | 37.30 | 45.70 |  |  |  |
| GSK2586881 i.v. 0.2 mg/kg | CVD-PAH (III) | 9 | CI (L/min/m2) | 5.10 |  |  | 5.30 | 5.60 | 4.70 |  |  |  |
| GSK2586881 i.v. 0.2 mg/kg | CVD-PAH (III) | 9 | PVR (Wood units) | 5.00 |  |  | 4.90 | 4.60 | 6.00 |  |  |  |
| GSK2586881 i.v. 0.4 mg/kg | Idiopathic PAH (II) | 10 | Ang II / Ang (1-7) | 2.64 | 0.67 | 0.81 | 0.27 | 0.12 | 0.10 | 0.07 | 0.81 | 1.50 |
| GSK2586881 i.v. 0.4 mg/kg | Idiopathic PAH (II) | 10 | mPAP (mmHg) | 63.70 |  |  | 50.30 | 53.70 | 59.00 |  |  |  |
| GSK2586881 i.v. 0.4 mg/kg | Idiopathic PAH (II) | 10 | CI (L/min/m2) | 1.90 |  |  | 2.00 | 1.90 | 1.80 |  |  |  |
| GSK2586881 i.v. 0.4 mg/kg | Idiopathic PAH (II) | 10 | PVR (Wood units) | 11.60 |  |  | 8.90 | 9.00 | 6.00 |  |  |  |
| GSK2586881 i.v. 0.4 mg/kg | Idiopathic PAH (III) | 11 | Ang II / Ang (1-7) | 17.00 | 0.66 | 0.13 | 0.14 | 0.37 | 0.40 | 0.23 | 1.84 | 8.75 |
| GSK2586881 i.v. 0.4 mg/kg | Idiopathic PAH (III) | 11 | mPAP (mmHg) | 30.70 |  |  | 27.30 | 31.00 | 29.00 |  |  |  |
| GSK2586881 i.v. 0.4 mg/kg | Idiopathic PAH (III) | 11 | CI (L/min/m2) | 2.50 |  |  | 2.70 | 2.90 | 2.40 |  |  |  |
| GSK2586881 i.v. 0.4 mg/kg | Idiopathic PAH (III) | 11 | PVR (Wood units) | 4.30 |  |  | 3.70 | 4.20 | 4.70 |  |  |  |
| GSK2586881 i.v. 0.4 mg/kg | Heritable PAH (II) | 12 | Ang II / Ang (1-7) | 11.50 | 0.48 | 0.52 | 1.50 | 0.54 | 0.49 | 0.31 | 1.27 | 12.80 |
| GSK2586881 i.v. 0.4 mg/kg | Heritable PAH (II) | 12 | mPAP (mmHg) | 26.00 |  |  | 28.30 | 25.00 | 24.30 |  |  |  |
| GSK2586881 i.v. 0.4 mg/kg | Heritable PAH (II) | 12 | CI (L/min/m2) | 2.20 |  |  | 3.80 | 2.20 | 2.30 |  |  |  |
| GSK2586881 i.v. 0.4 mg/kg | Heritable PAH (II) | 12 | PVR (Wood units) | 3.90 |  |  | 2.00 | 3.40 | 3.20 |  |  |  |
| GSK2586881 i.v. 0.4 mg/kg | CVD-PAH (II) | 13 | Ang II / Ang (1-7) | 3.74 | 4.23 | 19.30 | 5.81 | 1.50 | 1.50 | 1.50 | 1.50 | 1.50 |
| GSK2586881 i.v. 0.4 mg/kg | CVD-PAH (II) | 13 | mPAP (mmHg) | 38.70 |  |  | 35.30 | 28.70 | 38.00 |  |  |  |
| GSK2586881 i.v. 0.4 mg/kg | CVD-PAH (II) | 13 | CI (L/min/m2) | 4.00 |  |  | 3.50 | 2.70 | 2.90 |  |  |  |
| GSK2586881 i.v. 0.4 mg/kg | CVD-PAH (II) | 13 | PVR (Wood units) | 4.20 |  |  | 3.80 | 4.20 | 4.80 |  |  |  |
| GSK2586881 i.v. 0.4 mg/kg | CVD-PAH (III) | 14 | Ang II / Ang (1-7) | 1.50 | 7.08 | 1.50 | 1.50 | 1.50 | 1.50 | 1.50 | 1.50 | 1.50 |
| GSK2586881 i.v. 0.4 mg/kg | CVD-PAH (III) | 14 | mPAP (mmHg) | 35.30 |  |  | 37.00 | 36.30 | 38.30 |  |  |  |
| GSK2586881 i.v. 0.4 mg/kg | CVD-PAH (III) | 14 | CI (L/min/m2) | 3.10 |  |  | 2.70 | 2.70 | 2.50 |  |  |  |
| GSK2586881 i.v. 0.4 mg/kg | CVD-PAH (III) | 14 | PVR (Wood units) | 5.60 |  |  | 6.50 | 6.40 | 7.10 |  |  |  |
| GSK2586881 i.v. 0.8 mg/kg | Idiopathic PAH (II) | 15 | Ang II / Ang (1-7) | 2.74 | 0.40 | 0.23 | 0.28 | 0.28 | 0.36 | 0.17 | 0.49 | 1.46 |
| GSK2586881 i.v. 0.8 mg/kg | Idiopathic PAH (II) | 15 | mPAP (mmHg) | 40.70 |  |  | 44.70 | 45.70 | 48.30 |  |  |  |
| GSK2586881 i.v. 0.8 mg/kg | Idiopathic PAH (II) | 15 | CI (L/min/m2) | 2.10 |  |  | 2.00 | 2.10 | 2.20 |  |  |  |
| GSK2586881 i.v. 0.8 mg/kg | Idiopathic PAH (II) | 15 | PVR (Wood units) | 6.40 |  |  | 8.90 | 7.50 | 7.60 |  |  |  |
| GSK2586881 i.v. 0.8 mg/kg | Idiopathic PAH (III) | 16 | Ang II / Ang (1-7) | 8.75 | 0.31 | 0.40 | 0.33 | 0.27 | 1.61 | 0.22 | 0.30 | 16.38 |
| GSK2586881 i.v. 0.8 mg/kg | Idiopathic PAH (III) | 16 | mPAP (mmHg) | 22.70 |  |  | 20.30 | 21.30 | 18.70 |  |  |  |
| GSK2586881 i.v. 0.8 mg/kg | Idiopathic PAH (III) | 16 | CI (L/min/m2) | 2.90 |  |  | 3.00 | 3.40 | 2.60 |  |  |  |
| GSK2586881 i.v. 0.8 mg/kg | Idiopathic PAH (III) | 16 | PVR (Wood units) | 1.70 |  |  | 1.70 | 1.20 | 1.90 |  |  |  |
| GSK2586881 i.v. 0.8 mg/kg | Idiopathic PAH (III) | 17 | Ang II / Ang (1-7) | 3.88 | 1.33 | 0.31 | 1.50 | 0.65 | 1.50 | 0.13 | 0.70 | 5.34 |
| GSK2586881 i.v. 0.8 mg/kg | Idiopathic PAH (III) | 17 | mPAP (mmHg) | 44.70 |  |  | 47.70 | 52.30 | 51.00 |  |  |  |
| GSK2586881 i.v. 0.8 mg/kg | Idiopathic PAH (III) | 17 | CI (L/min/m2) | 2.40 |  |  | 2.50 | 2.70 | 2.40 |  |  |  |
| GSK2586881 i.v. 0.8 mg/kg | Idiopathic PAH (III) | 17 | PVR (Wood units) | 7.30 |  |  | 6.90 | 7.60 | 8.00 |  |  |  |
| GSK2586881 i.v. 0.8 mg/kg | Heritable PAH (II) | 18 | Ang II / Ang (1-7) | 5.11 | 0.55 | 0.44 | 0.30 | 0.37 | 0.32 | 0.35 | 2.47 | 18.10 |
| GSK2586881 i.v. 0.8 mg/kg | Heritable PAH (II) | 18 | mPAP (mmHg) | 31.00 |  |  | 30.00 | 25.00 | 34.70 |  |  |  |
| GSK2586881 i.v. 0.8 mg/kg | Heritable PAH (II) | 18 | CI (L/min/m2) | 2.60 |  |  | 2.50 | 2.40 | 3.00 |  |  |  |
| GSK2586881 i.v. 0.8 mg/kg | Heritable PAH (II) | 18 | PVR (Wood units) | 4.60 |  |  | 4.50 | 3.40 | 4.40 |  |  |  |
| GSK2586881 i.v. 0.8 mg/kg | Heritable PAH (II) | 19 | Ang II / Ang (1-7) | 6.97 | 0.82 | 0.35 | 1.11 | 0.57 | 1.17 | 0.24 | 1.67 | 6.16 |
| GSK2586881 i.v. 0.8 mg/kg | Heritable PAH (II) | 19 | mPAP (mmHg) | 31.00 |  |  | 35.00 | 34.30 | 34.70 |  |  |  |
| GSK2586881 i.v. 0.8 mg/kg | Heritable PAH (II) | 19 | CI (L/min/m2) | 2.90 |  |  | 3.00 | 3.10 | 3.00 |  |  |  |
| GSK2586881 i.v. 0.8 mg/kg | Heritable PAH (II) | 19 | PVR (Wood units) | 4.10 |  |  | 4.80 | 4.70 | 4.80 |  |  |  |
| GSK2586881 i.v. 0.8 mg/kg | Heritable PAH (III) | 20 | Ang II / Ang (1-7) |  | 1.50 | 1.50 | 1.50 | 1.50 | 9.87 | 1.50 | 1.50 | 2.58 |
| GSK2586881 i.v. 0.8 mg/kg | Heritable PAH (III) | 20 | mPAP (mmHg) | 45.70 |  |  | 48.70 | 49.30 | 51.70 |  |  |  |
| GSK2586881 i.v. 0.8 mg/kg | Heritable PAH (III) | 20 | CI (L/min/m2) | 2.40 |  |  | 2.50 | 2.40 | 2.40 |  |  |  |
| GSK2586881 i.v. 0.8 mg/kg | Heritable PAH (III) | 20 | PVR (Wood units) | 6.90 |  |  | 6.80 | 7.60 | 7.90 |  |  |  |
| GSK2586881 i.v. 0.8 mg/kg | Heritable PAH (III) | 21 | Ang II / Ang (1-7) | 1.50 | 1.50 | 1.50 | 1.50 | 1.50 | 1.50 | 1.50 | 0.31 | 9.14 |
| GSK2586881 i.v. 0.8 mg/kg | Heritable PAH (III) | 21 | mPAP (mmHg) | 39.30 |  |  | 40.30 | 42.70 | 42.70 |  |  |  |
| GSK2586881 i.v. 0.8 mg/kg | Heritable PAH (III) | 21 | CI (L/min/m2) | 3.00 |  |  | 2.80 | 2.90 | 3.00 |  |  |  |
| GSK2586881 i.v. 0.8 mg/kg | Heritable PAH (III) | 21 | PVR (Wood units) | 5.10 |  |  | 5.60 | 5.70 | 6.10 |  |  |  |
| GSK2586881 i.v. 0.8 mg/kg | CVD-PAH (II) | 22 | Ang II / Ang (1-7) | 1.50 | 1.50 | 1.50 | 1.50 | 1.50 | 1.50 | 1.50 | 3.23 |  |
| GSK2586881 i.v. 0.8 mg/kg | CVD-PAH (II) | 22 | mPAP (mmHg) | 35.00 |  |  | 25.70 | 26.30 | 33.00 |  |  |  |
| GSK2586881 i.v. 0.8 mg/kg | CVD-PAH (II) | 22 | CI (L/min/m2) | 2.80 |  |  | 2.60 | 2.60 | 2.50 |  |  |  |
| GSK2586881 i.v. 0.8 mg/kg | CVD-PAH (II) | 22 | PVR (Wood units) | 4.00 |  |  | 2.80 | 3.60 | 4.30 |  |  |  |

Ang, angiotensin; CI, cardiac index; CVD-PAH, PAH associated with collagen vascular disease; FC, functional class; mPAP, mean pulmonary arterial pressure; PAH, pulmonary arterial hypertension; PVR, pulmonary vascular resistance

**Supplementary Figure 1.** Effect of single dose GSK2586881 (0.1, 0.2, 0.4, and 0.8 mg/kg) on Ang II/Ang (1-7), mPAP, CI, and PVR in patients with idiopathic, heritable PAH, and PAH associated with collagen vascular disease.


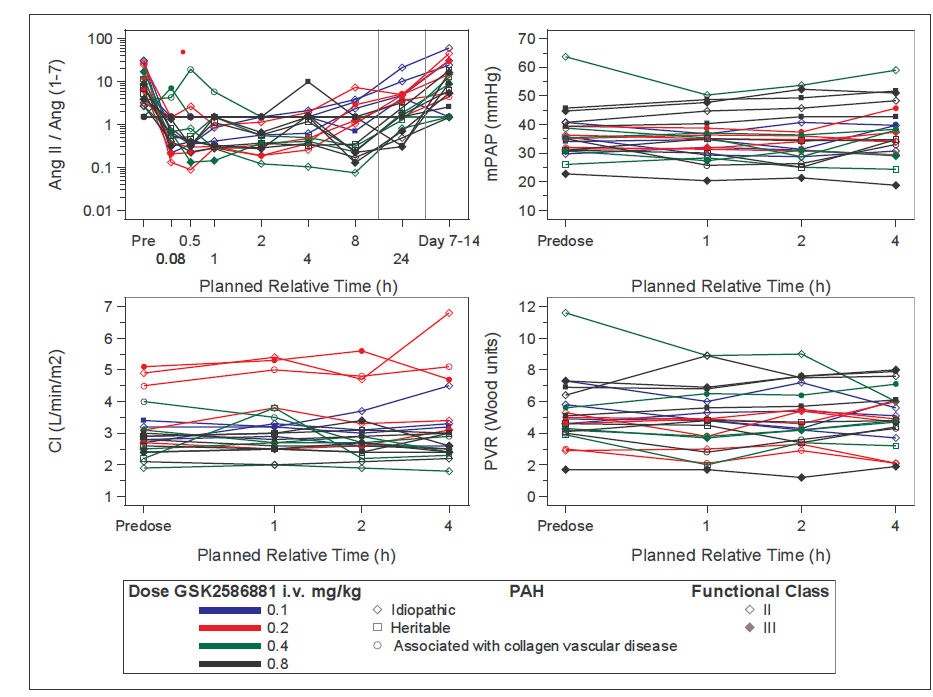


Ang, angiotensin; mPAP, mean pulmonary artery pressure; CI, cardiac index; PVR, pulmonary vascular resistance.

**SUPPLEMENTARY REFERENCES**

1. Gruenig E, Michelakis E, Vachiery JL, et al. Acute hemodynamic effects of single-dose sildenafil when added to established bosentan therapy in patients with pulmonary arterial hypertension: results of the COMPASS-1 study. *J Clin Pharmacol* 2009; 49: 1343-1352. 2009/09/17. DOI: 10.1177/0091270009341182.

2. Ghofrani HA, Voswinckel R, Reichenberger F, et al. Differences in hemodynamic and oxygenation responses to three different phosphodiesterase-5 inhibitors in patients with pulmonary arterial hypertension: a randomized prospective study. *J Am Coll Cardiol* 2004; 44: 1488-1496. 2004/10/07. DOI: 10.1016/j.jacc.2004.06.060.
