# Supplementary figures and images for: An open‐label, dose‐escalation study to evaluate the safety, tolerability, pharmacokinetics, and pharmacodynamics of single doses of GSK2586881 in participants with pulmonary arterial hypertension
Source: Pulm Circ. 2022 Jan 20;12(1):e12024. doi: 10.1002/pul2.12024 (PMC9053011; doi:10.1002/pul2.12024)

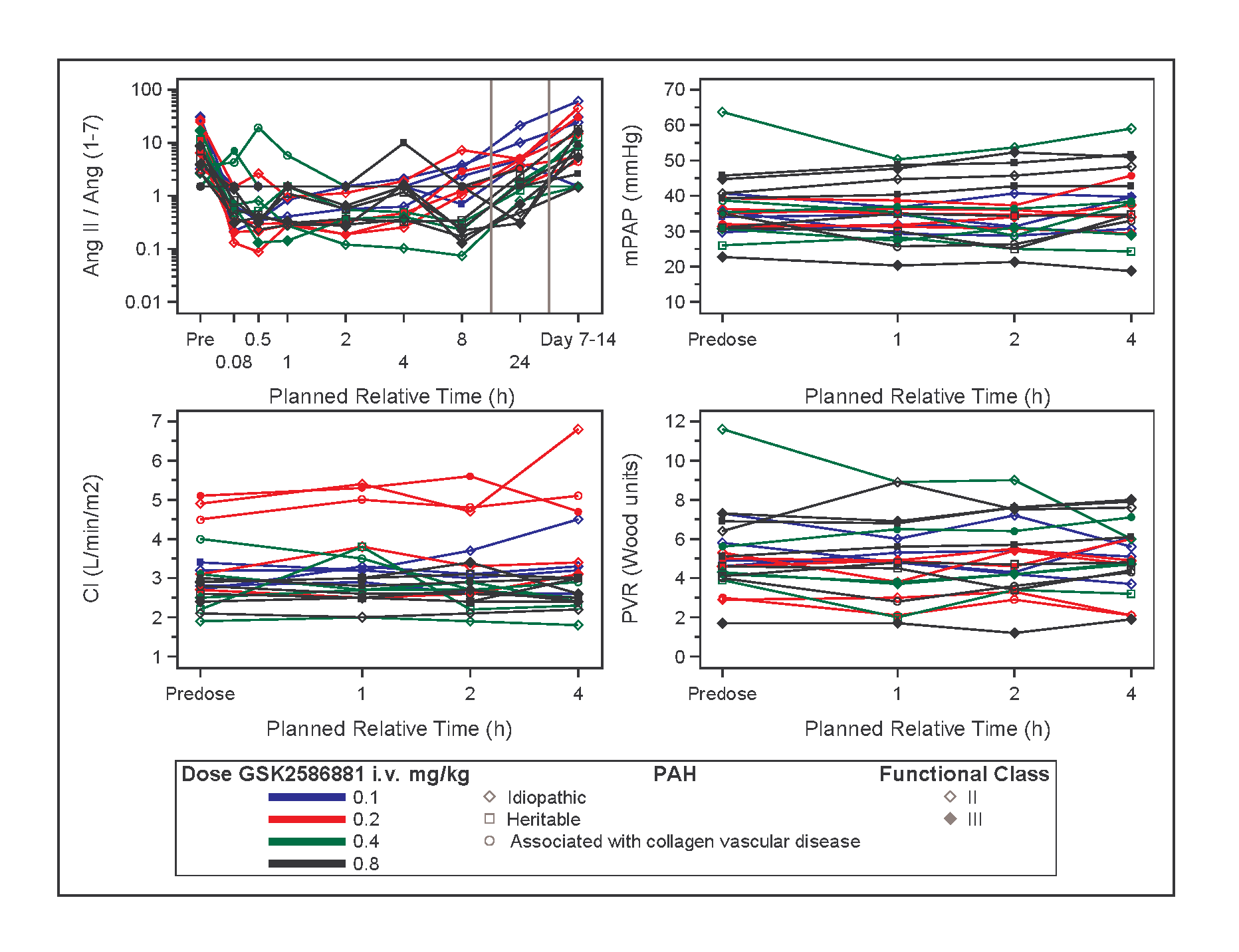

Supplement: Supplementary file 2 — Supporting information. [file PUL2-12-e12024-s001.tif]
